# Effect of optimal antenatal care on maternal and perinatal health in Ethiopia

**DOI:** 10.3389/fped.2023.1120979

**Published:** 2023-02-07

**Authors:** Neamin Tesfay, Girmay Hailu, Fitsum Woldeyohannes

**Affiliations:** ^1^ Center of Public Emergency Management, Ethiopian Public Health Institute, Addis Ababa, Ethiopia; ^2^ Health Financing Department, Clinton Health Access Initiative, Addis Ababa, Ethiopia

**Keywords:** optimal ANC care, uterine rupture, delay one, operative vaginal delivery, intrauterine life, Ethiopia

## Abstract

**Introduction:**

Receiving at least four antenatal care (ANC) visits have paramount importance on the health of mothers and perinates. In Ethiopia, several studies were conducted on ANC service utilization; however, limited studies quantified the effect of care on maternal and perinate health. In response to this gap, this study is conducted to quantify the effect of optimal ANC care (≥4 visits) on maternal and perinatal health among women who received optimal care in comparison to women who did not receive optimal care.

**Methods:**

The study utilized the Ethiopian perinatal death surveillance and response (PDSR) system dataset. A total of 3,814 reviewed perinatal deaths were included in the study. Considering the nature of the data, preferential within propensity score matching (PWPSM) was performed to determine the effect of optimal ANC care on maternal and perinatal health. The effect of optimal care was reported using average treatment effects of the treated [ATT].

**Result:**

The result revealed that optimal ANC care had a positive effect on reducing perinatal death, due to respiratory and cardiovascular disorders, [ATT = −0.015, 95%CI (−0.029 to −0.001)] and extending intrauterine life by one week [ATT = 1.277, 95%CI: (0.563–1.991)]. While it's effect on maternal health includes, avoiding the risk of having uterine rupture [ATT = −0.012, 95%CI: (−0.018 to −0.005)], improving the utilization of operative vaginal delivery (OVD) [ATT = 0.032, 95%CI: (0.001–0.062)] and avoiding delay to decide to seek care [ATT = −0.187, 95%CI: (−0.354 to −0.021)].

**Conclusion:**

Obtaining optimal ANC care has a positive effect on both maternal and perinatal health. Therefore, policies and interventions geared towards improving the coverage and quality of ANC services should be the top priority to maximize the benefit of the care.

## 1. Introduction

Antenatal care (ANC) is routine and regular maternity care provided for pregnant women from conception to the onset of labour (1). ANC is also considered as a precautionary measure for pregnant women to identify and manage pre-existing and potential causes of maternal and child health morbidity and mortality through supplementation of micronutrients, screening of infection, and health promotion (2, 3).

Globally, 87% of pregnant women had received ANC service at least once, by health professionals. However, not all women completed the bare minimum number of ANC visits (having at least four ANC visits). According to a similar study, only two-thirds (65%) of pregnant women receive four ANC visits. The proportion of complete ANC visits is different from region to region, Sub Sahara African and south Asian countries have the lowest proportion at 52% and 48% respectively (4). Ethiopia has made remarkable progress in improving the coverage of ANC service in the last two decades (5). The coverage has increased from 27% in 2000 to 74% in 2019. In line with this, the number of women who attend four and more ANC visits (ANC 4+) has also increased from 10% in 2000 to 43% in 2019 (5, 6). Similarly, the proportion of women who began ANC visits within the recommend period [between 8 and 12 gestational weeks] has also increased from 6% in 2000 to 28% in 2019 (5–7).

World Health Organization (WHO) has made continuous improvements to the protocol of ANC visits, and one of the recent recommendations was to increase the minimum number of ANC visits from four contacts to eight contacts (8). However, Ethiopia was stacked with the previous protocol of focused ANC care till 2021; however, lately, the country has accepted and adopted the recent recommendation of positive pregnancy experience for ANC visits in 2022 (9).

Ethiopia has taken various measures to address the barriers related to ANC service utilization. Those barriers are broadly classified into three categories: namely, perception, access, and quality (10). In response to this, the country has introduced a health extension program, and community-based health insurance to improve the service uptake. In addition, community health structures were organized to boost community engagement and awareness in the health system (11–13). Ensuring sustainable health financing and enacting measures aimed at installing quality care improvement (QIA) were some of the steps taken to improve access and quality of care (14, 15). Despite making the aforementioned efforts, the coverage and quality of the care remain unsatisfactory (9). The quality of care is compromised due to the lack of trained personnel, medical equipment, and physical health infrastructure (16–19). In addition, the presence of noticeable regional variation, lack of an effective monitoring mechanism for the proposed intervention, and lack of updated and data-driven evidence for decision-making were major barriers to realizing the target ascertained in the national strategy (20–23).

To address the gap in evidence generation and to produce a data-driven policy recommendation, The country has established maternal and perinatal death surveillance and response (MPDSR) system, to obtain updated information on the cause and contributing factors related to preventable maternal and perinatal death (24). As a result, both maternal and perinatal death are included under the surveillance system as compulsory events to be reported to the next level (25). ANC service utilization was included in the surveillance system as one component to evaluate and review maternal and perinatal death concerning ANC history (26). However, the system was not fully implemented due to the presence of limited community engagement, lack of trained personnel, lack of maternal health infrastructure, fear of legal repercussions, and defensive attitude towards the system (27, 28).

Having a proper ANC visit has a tremendously positive effect on the health of the mother and her child in addition to enhancing health promotion (29). Some of the benefits of complete ANC care on maternal health include reducing the probability of having maternal near miss, postpartum hemorrhage, preterm labour, and anemia (30–32). While dropping the risk of stillbirth, declining neonate death, improving the nutritional status of children, and reducing neonatal admission to neonatal intensive care (NICU) were included under neonatal positive effects of ANC care (33–38). Improving exclusive Breastfeeding practice, avoiding pre-lacteal feeding, enhancing the utilization of postnatal care, encouraging institutional delivery, augmenting the uptake of essential new care services, boosting the adherence to iron folate supplementation, ameliorating full vaccination of children, and increasing awareness of the danger sign of neonates are some major benefits in health promotion aspect (39–45). Considering all the health benefits stated above, the Ethiopian Federal Ministry of Health (FMOH) selected ANC as a priority area of research under the domain of improving maternal and child health (46).

Several studies were carried out, in Ethiopia, through the conventional regression analysis approach to assess the benefit of ANC care utilization without controlling potential confounders in the analysis (47–49). However, propensity score analysis (PSA) offers an alternative approach for program evaluation in a situation where randomized controlled trials are either infeasible or unethical through observational data (50). PSA has the potential to handle the drawback of conventional regression, which is the risk of bias on the exposures due to unobservable characteristics in the data set (51). PSA reduces this bias by matching women who attended 4 or more ANC visits (exposed) and those who attended less than 4 ANC visits (unexposed) with similar conditional probabilities to receive the treatment. The adjustment on the potential confounder makes PSA preferable to the traditional regression (52). The utilization of propensity score (PS) helps to make a balanced score within the potential cofounder between the exposed and unexposed groups based on surveillance data, hence resembling characteristics of randomized trials (53). To have a comparable balanced group of the respondent for the observed covariates, potential factors that influenced the utilization of ANC such as maternal age, maternal parity, maternal education status, religion, residence, ownership of the facility, type of health facility, and type of region were balanced accordingly (54–58). Considering the gaps in previous studies, and the benefit of the proposed approach, PSA was employed to determine the effect of optimal ANC care on maternal and perinatal health.

## 2. Methods

### 2.1. Study setting

Ethiopia has an estimated population of 117,876,000 in 2021, out of which 3,772,032 are under-one-year children (59). Administratively, Ethiopia has ten regions and two city administrations, namely Tigray, Afar, Amhara, Oromia, Somali, Benishangul-Gumuz, Southern Nations Nationalities, and Peoples Region (SNNPR), Sidama, Gambella, Harari, Addis Ababa city administration and Dire Dawa city administration (60). In 2020/21, there were 434 hospitals, 3,890 health centers, and 18,090 health posts with a health work density of 3.2 per 100,000 population (61). The country has high infant, under-five, and maternal mortality (47 per 1,000 LBs), (59 per 1,000 LBs), and (412 per 10,000 LBs), respectively (5, 6).

### 2.2. Data source and study participants

Epidemiological review of perinatal death was obtained from the Ethiopian Public Health Institutes (EPHI) during the four consecutive years of implementation (2018–2021). The data is collected and compiled from various health facilities across the country. The source population for this study is all perinate who died and were reviewed by the MPDSR committee during the study period. The system used facility-based abstraction format (FBAF) and verbal autopsy (VA) as data sources for the review process. Following the data extraction from the source, a review committee discuss and prepare the final report of the reviewed death. The final report will then be sent to the national data hub through perinatal death reporting format (PDRF) by the focal person assigned in each reporting facility. A total of 3,814 reviewed perinatal deaths were included in the study.

### 2.3. Study variables

Treatment (T) = 1 if woman had four or more ANC visits during the time of pregnancy; whereas women who had less than four ANC visits were labeled as “0”.

### 2.4. Outcome variables

Individual (maternal and perinatal) variables were selected after an extensive search of various works of literature. Thus, gestation age, place of delivery, cause of perinatal death, and mode of delivery were selected as perinatal variables, while maternal health condition, maternity outcome, and a score of delay one (delay to seek care) were considered as maternal health indicators. Cause of perinatal death and maternal health conditions were classified based on the International Classification of Diseases -Perinatal Mortality (ICD-PM) (62).

The score of delay one, which is a delay in deciding to seek care (63), was computed using the row sum of seven variables included under the domain; namely (1) family poverty, (2) bad experience with previous health care, (3) failed to recognize the danger signs of pregnancy,4) unaware where to go, (5) had no one take care of other children, (6) reliant on traditional practice and (7) lack of decision to a health facility. All of them were binary variables with “Yes” and “No” responses and after summation of the score and to keep the normality of the data a square root transformation was done (64). Finally, the transformed variable was treated as continuous variables to make a simple and parsimonious model (65).

### 2.5. Operational definition

#### 2.5.1. Type of facility

Classified into three classes (primary, secondary, and tertiary health care facilities) according to their manpower, medical equipment, and service provision (61).

#### 2.5.2. Optimal ANC visit

Women who had four and more ANC visits between conception up to the delivery of the perinate (66).

The cause of perinatal death was categorized by the time of death; antepartum, intrapartum stillbirth, unknown time of stillbirth, and neonatal death. The contributing maternal conditions were classified into five major categories (M1 to M4, with M5 representing no identified condition) per the guidance of ICD_PM_10 (Table 1) (67).

**Table 1:** ICD-PM categories with the specific cause of perinatal death and maternal health condition.

Time of death	Category	Description	Example
Antepartum death	A1	Congenital malformations and chromosomal abnormalities	Anencephaly, encephalocele, microcephaly, congenital hydrocephalus, spina bifida, etc.
	A2	Infection	Congenital syphilis, congenital malaria, congenital rubella syndrome, congenital TB, etc.
	A3	Antepartum hypoxia	Intrauterine hypoxia
	A4	Other specified antepartum disorder	Vasa previa, ruptured cord, twin-twin transfusion, Intraventricular (nontraumatic) haemorrhage, Rhesus and ABO isoimmunization, etc.
	A5	Disorders related to fetal growth	Small for gestational age, macrosomia, post-term, etc.
	A6	Antepartum death of unspecified cause	Intrauterine death of unspecified cause
Intrapartum death	I1	Congenital malformations and chromosomal abnormalities	Anencephaly, encephalocele, microcephaly, congenital hydrocephalus, spina bifida, etc.
	I2	Birth trauma	Intracranial laceration and haemorrhage due to birth injury, Fracture of skull due to birth injury, etc.
	I3	Acute intrapartum event	Intrauterine hypoxia
	I4	Infection	Congenital syphilis, congenital malaria, congenital rubella syndrome, congenital TB, etc.
	I5	Other specified intrapartum disorder	Vasa previa, ruptured cord, twin-twin transfusion, Intraventricular (nontraumatic) haemorrhage, Rhesus and ABO isoimmunization, etc.
	I6	Disorders related to fetal growth	Small for gestational age, extremely low birth weight, macrosomia, post-term, etc.
	I7	Intrapartum death of unspecified cause	Fetal death of unspecified cause
Neonatal death	N1	Congenital malformations, and chromosomal abnormalities	Anencephaly, encephalocele, microcephaly, congenital hydrocephalus, spina bifida, etc.
	N2	Disorders related to fetal growth	Small for gestational age, exceptionally large baby, post-term, etc.
	N3	Birth trauma	Cerebral haemorrhage due to birth injury, intraventricular haemorrhage due to birth injury, etc.
	N4	Complications of intrapartum events	Intrauterine hypoxia, birth asphyxia
	N5	Convulsions and disorders of cerebral status	Neonatal cerebral irritability, neonatal cerebral depression, neonatal coma, etc.
	N6	Infection	Tetanus neonatorum, bacterial meningitis, bacterial sepsis, congenital pneumonia, etc.
	N7	Respiratory and cardiovascular disorders	Respiratory distress syndrome, Neonatal aspiration syndromes, neonatal cardiac failure, neonatal cardiac dysrhythmia, neonatal hypertension, etc.
	N8	Other neonatal conditions	Vasa previa, ruptured cord, twin-twin transfusion, Rhesus and ABO isoimmunization, kernicterus, etc.
	N9	Low birthweight and prematurity	Extremely low birth weight, extreme immaturity
	N10	Miscellaneous	Cases where codes from several other sections of ICD-10 should be used
	N11	Neonatal death of unspecified cause	Congenital renal failure, termination of pregnancy, affecting fetus and newborn, withdrawal symptoms from drug
Maternal conditions	M1	Complications of placenta, cord and membranes	Abruptio placentae, prolapsed cord, chorioamnionitis, etc.
	M2	Maternal complications of pregnancy	Premature rupture of membranes, oligo- and polyhydramnios, ectopic pregnancy, multiple pregnancy, etc.
	M3	Other complications of labour and delivery	Breech delivery and extraction, forceps delivery, Caesarean delivery, Uterine rupture
	M4	Maternal medical and surgical conditions	hypertensive disorders, maternal injury, maternal use of tobacco, alcohol or drugs, etc.
	M5	No maternal conditions	No condition identified

### 2.6. Data management and statistical analysis

For data cleaning and further analysis, the data was exported from Epi -info version 7.2 to Stata version 17, and both descriptive and analytical analyses were carried out. The descriptive analysis tables were presented using two-way tables, by classifying the table using the level of ANC care and whether the women received optimal ANC care or not. The analytical analysis was carried out using preferential within propensity score matching (PWPSM).

### 2.7. Model building strategy

To identify the final effect of treatment on the study outcome a series of statistical procedures were conducted. The following modeling building strategy was followed in estimating treatment effects which are: (1) “Estimating propensity score”, (2) “stratifying and balancing propensity score”, and (3) “estimating causal effect” (68, 69). Proper matching was also conducted since it is practically impossible to see the effect of optimal care on mothers and perinates without it.

#### 2.7.1. Step one

The prosperity score (PS) was estimated for each observation, which indicates the probability of the individual unit experiencing the treatment (in this case receiving optimal ANC care). The score is computed using the potential individual and community level factors, which had a substantial role in the uptake of ANC.

#### 2.7.2. Step two

After computing PS, the balance check was performed to look through the score if it is biased or not. As indicated in Supplementary Annex S1, the standardized biased was observed across the covariates with a range of −40 to 10.

#### 2.7.3. Step three

To address the observed bias across the covariates, matching was performed using the nearest neighbor caliber matching with a radius of 0.25 standard deviation of the PS. After this arrangement, matching was carried out to avoid the bias observed in Supplementary Annex S1. As displayed in Supplementary Annex S2, a balance check of the PS was carried out to look at the quality of the matching, standardized bias was within an acceptable range of −10 to 10.

#### 2.7.4. Step four

Selected an appropriate method of matching in considering the nature of the data. The surveillance data was hierarchal., i.e., the mother and deceased perinate were nested in 161 reporting health facilities and 45 provinces of the country. The data had both small and big clusters (reporting health facility) and by considering the nature of the cluster preferential within propensity score matching (PWPSM) was preferable and selected for the final PSA (70).

#### 2.7.5. Step five

After model selection, the effect of the treatment was reported through the average treatment effect of treated (ATT) of the given covariate on selected outcome maternal and perinatal health interest (71).

## 3. Result

### 3.1. Selected characteristics of the reported facility

A total of 3,814 deceased perinates and their mothers were included in the study. Among mothers of the deceased perinate, only 33.9% of mothers received optimal ANC visits during their pregnancy. Among reporting facilities, 51.5% of mothers who were treated in tertiary health facilities received optimal ANC care. Region-wise, the proportion of mothers who received optimal ANC care was higher among mothers of a deceased perinate who resided in Addis Ababa (51.9%) than the mother of a deceased perinate who resided in the Gambella region (0.0%) (Table 2).

**Table 2:** Selected background characteristics of reporting facilities by the level of ANC follow-up in Ethiopia, 2021.

Characteristic	Not optimal, *N *= 2,518	Optimal visit, *N* = 1,296	Overall, *N* = 3,814
**Type of health facility**			
Primary health care	1,406 (70.3%)	593 (29.7%)	1,999
Secondary health care	658 (74.9%)	220 (25.1%)	878
Tertiary health care	454 (48.5%)	483 (51.5%)	937
**Facility ownership**			
Public facility	2,515 (66.2%)	1,286 (33.8%)	3,801
Non-governmental organization	0 (0.0%)	2 (100.0%)	2
Private	3 (27.3%)	8 (72.7%)	11
**Data source**			
FBAF	2,390 (65.7%)	1,249 (34.3%)	3639
VA	128 (73.1%)	47 (26.9%)	175
**Reporting region**			
Addis Ababa	389 (48.1%)	419 (51.9%)	808
Amhara	1,316 (66.2%)	673 (33.8%)	1,989
Benishangul Gumuz	58 (80.6%)	14 (19.4%)	72
Dire Dawa	29 (76.3%)	9 (23.7%)	38
Gambella	4 (100.0%)	0 (0.0%)	4
Harir	15 (71.4%)	6 (28.6%)	21
Oromia	464 (81.7%)	104 (18.3%)	568
Sidama	90 (93.8%)	6 (6.2%)	96
SNNPR	94 (61.0%)	60 (39.0%)	154
Somali	59 (92.2%)	5 (7.8%)	64
**Year of reporting**			
2018	248 (55.4%)	200 (44.6%)	448
2019	448 (57.3%)	334 (42.7%)	782
2020	639 (72.7%)	240 (27.3%)	879
2021	1,183 (69.4%)	522 (30.6%)	1,705

### 3.2. Selected characteristics of the mothers of the deceased perinate

The average maternal parity among mothers of deceased perinate was higher among mothers who did not receive optimal ANC care (2.4(with SD of 1.79) than mothers who received an optimal ANC visit (2.3(with SD of 1.62). The proportion of mothers who received optimal ANC care was higher among mothers who attend secondary education and above (47.9%) than mothers who had no education (27.1%). The proportion of mothers with a deceased perinate who received optimal care was higher among mothers who live in urban areas (41.1%) than mothers who live in rural areas (28.1%). Furthermore, women with no identified maternal complications had a higher proportion of receiving optimal ANC care (35.9%) than women with maternal complications of pregnancy (23.9%) (Table 3).

**Table 3:** Selected background characteristics of the deceased perinate's mother by the level of follow-up in Ethiopia, 2021.

Characteristic	Not optimal, *N* = 2,518	Optimal visit, *N* = 1,296	Overall, *N* = 3,814
**Maternal age** ^a^	27.2 (5.4)	27.6 (5.4)	27.3 (5.4)
**Maternal parity** ^a^	2.44 (1.79)	2.31 (1.62)	2.40 (1.73)
**Maternal educational status**			
Illiterate	1,581 (72.9%)	587 (27.1%)	2,168
Primary	570 (60.6%)	371 (39.4%)	941
Secondary and above	367 (52.1%)	338 (47.9%)	70
**Religion of the mother**			
Christian	1,919 (64.4%)	1,059 (35.6%)	2,978
Muslim	580 (71.4%)	232 (28.6%)	81
Traditional	19 (79.2%)	5 (20.8%)	24
**Residence**			
Urban	1,010 (58.9%)	706 (41.1%)	1,716
Rural	1,508 (71.9%)	590 (28.1%)	2,098
**Maternal health condition**			
Complications of placenta, cord, and membranes	282 (72.5%)	107 (27.5%)	389
Maternal complications of pregnancy	248 (76.1%)	78 (23.9%)	326
Maternal medical and surgical conditions	191 (64.7%)	104 (35.3%)	295
No maternal conditions identified	1,707 (64.1%)	957 (35.9%)	2,664
Other complications of labour and delivery	90 (64.3%)	50 (35.7%)	140
**Maternity outcome**			
Alive	2,164 (64.5%)	1,189 (35.5%)	3,353
Died	354 (76.8%)	107 (23.2%)	461

^a^ Mean (SD).

### 3.3. Selected characteristics of the deceased perinate

The average gestational week of the perinate was higher among mothers who received optimal ANC care (36.1(with SD of 3.5) than mothers who did not receive optimal ANC care (35.2(with SD of 3.4). The proportion of mothers who received optimal ANC care was higher among mothers who give birth at health facilities (35.5%) than mothers who give birth at home (15.5%). Furthermore, the proportion of mothers who received optimal ANC care was higher among mothers who deliver through cesarean section (46.5%) than mothers who deliver through Spontaneous vaginal delivery (31.4%) (Table 4).

**Table 4:** Selected characteristics of the deceased perinate by the level of ANC follow-up in Ethiopia, 2021.

Characteristic	Not optimal, *N* = 2,518	Optimal visit, *N* = 1,296	Overall, *N* = 3,814
**Estimated gestational age** ^a^	35.2 (3.38)	36.1 (3.52)	35.5 (3.45)
**Sex**			
Female	1,096 (67.9%)	518 (32.1%)	1,614
Male	1,422 (64.6%)	778 (35.4%)	2,200
**Place of birth**			
Health facility	2,244 (64.5%)	1,234 (35.5%)	3,478
Home	224 (84.5%)	41 (15.5%)	265
On transit	50 (70.4%)	21 (29.6%)	71
**Mode of delivery**			
Cesarean section	278 (53.5%)	242 (46.5%)	520
Operative vaginal delivery	134 (60.1%)	89 (39.9%)	223
Spontaneous vaginal delivery	2,106 (68.6%)	965 (31.4%)	3,071
**Place of death**			
Health facility	2,158 (64.5%)	1,190 (35.5%)	3,348
Home	281 (79.4%)	73 (20.6%)	354
On Transit	79 (70.5%)	33 (29.5%)	112
**Timing of death**			
Antepartum	134 (66.0%)	69 (34.0%)	203
Intrapartum	462 (69.4%)	204 (30.6%)	666
Neonatal	1,478 (61.7%)	919 (38.3%)	2,397
Unknown time stillbirth	444 (81.0%)	104 (19.0%)	548

^a^ Mean (SD).

### 3.4. Assigned cause of death

The proportion of perinatal death due to disorders related to fetal growth was higher among mothers who received optimal ANC care (87.7%) than mothers who did not receive optimal ANC care (12.4%). The proportion of perinatal death due to Acute intrapartum events was higher among mothers who received optimal ANC care (73.5%) than mothers who did not receive optimal ANC care (26.5%). Furthermore, the proportion of perinatal death due to infection events was higher among mothers who received optimal ANC care (73.5%) than mothers who did not receive optimal ANC care (26.5%) (Table 5).

**Table 5:** Assigned cause of death by the level of ANC follow-up in Ethiopia, 2021.

Characteristic	Not optimal, *N* = 2,518	Optimal visit, *N* = 1,296	Overall, *N* = 3,814
**Assigned cause of death**			
Antepartum death of unspecified cause	0 (0.0%)	1 (100.0%)	1
Other specified antepartum disorder	1 (50.0%)	1 (50.0%)	2
Intrapartum death of unspecified cause	2 (100.0%)	0 (0.0%)	2
Convulsions and disorders of cerebral status	4 (100.0%)	0 (0.0%)	4
Birth trauma	10 (55.6%)	8 (44.4%)	18
Acute antepartum event	14 (56.0%)	11 (44.0%)	25
Other neonatal conditions	15 (60.0%)	10 (40.0%)	25
Respiratory and cardiovascular disorders	76 (69.1%)	34 (30.9%)	110
Congenital malformations, deformations, and chromosomal	161 (60.3%)	106 (39.7%)	267
Disorders related to fetal growth	240 (87.6%)	34 (12.4%)	274
Acute intrapartum event	249 (73.5%)	90 (26.5%)	339
Complications of intrapartum events	393 (57.6%)	289 (42.4%)	682
Low birth weight and prematurity	620 (65.5%)	326 (34.5%)	946
Infection	733 (65.5%)	386 (34.5%)	1,119

### 3.5. Delay factor by the level of ANC care

The proportion of perinatal death contributed by delay one was higher among mothers who received optimal ANC care due to previous bad experiences in health facilities (36.4%) than mothers who delayed seeking care due to relying on traditional remedies (13.5%). The proportion of perinatal death contributed by delay two was higher among mothers who received optimal ANC care due to the expensive cost of transportation (39.6%) than mothers who delayed reaching the health facility due to poor road conditions (24.3%). Furthermore, the proportion of perinatal death contributed by delay three was slightly higher among mothers who received optimal ANC care due to the wrong diagnosis and treatment (56.3%) than mothers who were delayed to receive the care due to multiple referrals (42.8%) (Table 6).

**Table 6:** Delay factors by the level of ANC follow-up in Ethiopia, 2021.

Characteristic	Not optimal, *N* = 2,518	Optimal visit, *N* = 1,296	Overall, *N* = 3,814
**Delay 1 – Decision to seek care**			
Family poverty	195 (75.9%)	62 (24.1%)	257
Bad experience with previous health care	7 (63.6%)	4 (36.4%)	11
Failed to recognize the danger of pregnancy	813 (73.6%)	291 (26.4%)	1,104
Unaware where to go	42 (77.8%)	12 (22.2%)	54
Had no one take care of other children	37 (74.0%)	13 (26.0%)	50
Reliant on traditional practice	32 (86.5%)	5 (13.5%)	37
Lack of decision on a health facility	331 (72.9%)	123 (27.1%)	454
**Delay 2 – Reaching care**			
Absence of transportation	158 (69.6%)	69 (30.4%)	227
Expensive cost of transportation	32 (60.4%)	21 (39.6%)	53
No facility within a reasonable distance	92 (73.0%)	34 (27.0%)	126
Poor road condition	78 (75.7%)	25 (24.3%)	103
**Delay 3 – Receiving care**			
Long travel time from health facility to health facility	435 (57.2%)	325 (42.8%)	760
Long waiting time before treatment was received	179 (56.1%)	140 (43.9%)	319
Mistaken during an assessment, diagnosis, and treatment	38 (43.7%)	49 (56.3%)	87
Shortage of equipment and supplies	125 (56.8%)	95 (43.2%)	220

### 3.6. Propensity scores matching analysis

After matching and considering the cluster effect of the data, the probability of perinatal death due to respiratory and cardiovascular disorder among perinates born from mothers who received optimal ANC care was 2% [ATT = −0.015, 95%CI: (−0.029 to −0.001)] lower as compared to perinates who were born from the same mother who did not receive optimal ANC care. Furthermore, perinates who were born from mothers who received optimal ANC care had 1 week [ATT = 1.277, 95%CI: (0.563–1.991)] additional intrauterine life as compared to perinate who were born from the same mother who received less than four ANC visits. The likelihood of having uterine rupture among mothers who received optimal ANC care was 1% [ATT = −0.012, 95%CI: (−0.018 to −0.005)] lower as compared to the same women who did not receive optimal ANC care. The probability of giving birth through operative vaginal delivery (OVD) was 3% [ATT = 0.032, 95%CI: (0.001–0.062)] higher among mothers who received optimal ANC care as compared to the same mother who received less than four ANC visits. Additionally, the probability of delay to decide to seek care was 20% [ATT = −0.187, 95%CI: (−0.354 to −0.021)] lower among mothers who received optimal ANC care as compared to the same mothers who did not receive optimal ANC care (Table 7).

**Table 7:** Average treatment effects on the treated (ATT) 4 and more ANC visits on maternal and perinatal health in Ethiopia, 2021.

Variables	ATT	Standard error	95% CI		*P*-value
Respiratory and cardiovascular disorders	−0.015	0.007	−0.029	−0.001	0.039
Estimated Gestational week	1.277	0.361	0.563	1.991	0.001
Uterine rupture	−0.012	0.003	−0.018	−0.005	0.001
Operative vaginal delivery	0.032	0.015	0.001	0.062	0.041
A score of delay one	−0.187	0.084	−0.354	−0.021	0.028

## 4. Discussion

The study aimed to quantify the effect of optimal ANC care on maternal and perinatal health. Per the final model optimal ANC care had both perinatal (gestational week and respiratory and cardiovascular disorders) and maternal (uterine rupture, operative vaginal delivery, and delay one) health outcomes.

Perinates who were born from mothers who received optimal ANC care had a lower chance of dying due to respiratory and cardiovascular disorders as compared to perinate who were born from mothers who did not receive optimal ANC visits. The finding was supported by the study conducted elsewhere (72, 73). This could be explained by the fact that mothers who received optimal ANC care had a better opportunity than mothers who do not receive the optimal care, in identifying and managing the potential predisposing factors leading to complications of respiratory and cardiovascular disorders. In Ethiopia, respiratory distress syndrome and neonatal aspiration syndromes are the commonest cause of perinate death under the domain of respiratory and cardiovascular disorders (74, 75). Uncontrolled gestational diabetes Mellitus (GDM), pregnancy-induced hypertension (PIH), short birth interval, home delivery, and preterm delivery are identified as modifiable risk factors for respiratory and cardiovascular disorders in Ethiopia (74–76). These predisposing factors could be managed by adhering to the protocol of ANC follow-up, which encourages dietary diversity and physical activity to reduce the risk of GDM and PIH (77, 78) and boost the utilization of postpartum contraceptives to improve the birth interval between consecutive pregnancies (79). On top of this, receiving optimal ANC care improves institutional delivery, which paves the way to access the best treatment modalities for managing respiratory and cardiovascular disorders including antenatal corticosteroids, surfactants, and advanced respiratory care (80). However, the availability of those advanced treatment modalities is limited in a resource-constrained setting (81). In response to this gap, Ethiopia has taken initiative in equipping selected health facilities with grade three neonatal intensive care equipment (82). Overall, the finding implied that optimal ANC care has a positive impact in reducing the death of perinates due to respiratory and cardiovascular disorders.

Perinates who were born from mothers who received optimal ANC care had one additional week of intrauterine life as compared to perinate who were born from mothers who did not receive optimal ANC visits. This indicated that receiving optimal ANC care had a role in the reduction of preterm delivery. The finding was comparable with studies conducted in Uganda (83), Bangladesh (84), Surinam (85), and Brazil (86). This might be explained by the role of optimal ANC care in creating a convenient environment to take precautionary measures such as obtaining multiple micronutrient supplementation, HIV treatment, adequate malaria prophylaxis, and counseling on a healthy lifestyle (87–90). In Ethiopia, experiencing premature rupture of the membrane (PROM), PIH, being HIV positive, urinary tract infection, and short birth interval were known modifiable factors prone to preterm delivery (91–93). In line with this, respiratory distress syndrome was the major cause of death among preterm perinate born in Ethiopia (94). Optimal ANC care enhances the utilization of postpartum contraceptives, obtaining nutritional supplementation, receiving pharmacological therapy, and conducting routine urine tests. All these measures reduce the risk of having preterm birth by improving birth interval, managing complications of PIH, and treating urinary tract infections (95–97). In general, the finding implied that receiving optimal ANC care could reduce the risk of having preterm delivery and complications that comes up with preterm delivery.

Women who received optimal ANC care had a lower risk of encountering uterine rupture as compared to the same women who did not receive optimal ANC care. The finding was aligned with studies conducted in different parts of Ethiopia (Adama, Dessie, Mizan Tepi, and Tigray) (98–101). This could be explained by optimal ANC's role in the identification of potential risk exposure to uterine rupture and bolster for immediate and planned intervention such as cesarean section(C/S) (102). Home delivery, obstructed labour, prolonged labour, severe anemia, previous history of C/S, and partograph utilization were identified as risk factors for uterine rupture in Ethiopia (103–105). All those risk factors are handled during ANC visits by providing iron folate, encouraging institutional delivery, and planning selective C/S (106, 107). Overall, the findings suggested that optimal ANC visits had an integral role in reducing the risk of having a uterine rupture. Hence, the effect of optimal visits should be maximized well by integrating it into other health programs for better maternal and perinatal outcomes.

The study also elucidated that those women who received optimal ANC care had a high probability of giving birth through operative vaginal delivery (OVD) as compared to similar women who attend less than 4 ANC visits. The finding was coherent with a study conducted in a different part of Ethiopia (Jimma and Hossana) (108, 109). The plausible explanation could be the fact that OVD is one of the packages in basic emergency obstetric care provided in health facilities, which is designed to shorten second-stage labour through the assistance of forceps and vacuums (110). On top of this, OVD is solely provided in the health facility by a trained health care provider, which makes it more accessible for women who received optimal ANC since they have a higher probability of having institutional delivery, compared to women who do not receive optimal care (111). The presence of fetal distress, prolonged stage of labour, maternal health condition (maternal cardiac disease and PIH), and previous C/S were the common indications of OVD in Ethiopia (112). However, the OVD procedure unless taken meticulously could result in unwanted maternal and child outcomes such as a post-partum hemorrhage to the mother and cerebral palsy for the neonate (113, 114). Overall, the finding suggested that receiving optimal ANC had a role in increasing the chance of obtaining basic obstetrics emergency services.

Receiving optimal ANC had a substantial role in avoiding delay one, which is a delay in deciding to seek care. The finding corresponded well with studies conducted in Ethiopia (Hawassa, Shashemene, South Gonder, and Jimma) (115–118) and Mali (119). This could be explained by the better birth preparedness that those who received optimal ANC have, making them more alert and ready for pregnancy-related emergencies (120). Delay in deciding to seek care is more common in women who live in rural areas with low educational status (121). Furthermore, the delay vitiated the completion of the continuum of maternity care (47). Considering all this gap, Ethiopia has designed a health extension program to provide basic health education in a way to improve the health-seeking behaviors of the rural community (122). In addition, community engagement and creating a woman-friendly environment were some of the measures taken to improve the utilization and quality of ANC care. To this effect, the coverage of ANC has remarkably improved in the last two decades (9). Overall, the finding implied that ANC could be used as one platform to enhance community health-seeking behaviors in coordination with other parallel programs.

The study had the following limitations that needs to be acknowledged. Firstly, almost all reviewed death come from the public facility with limited involvement from a private facility, which affects the inclusiveness of the study. Secondly, the surveillance system captured a small number of deaths as compared to the national estimate, which also affect the representativeness of the study.

## 5. Conclusion

The study has identified that optimal ANC care affects maternal and perinate health. Reduced risk of death due to respiratory and cardiovascular disorders and lower preterm birth were effects included under perinatal health, while reduced the risk of having uterine rupture, enhancing birth giving through OVD, and improving the health-seeking behavior of women were included under maternal health. Thus, an exerted effort is needed in improving the coverage and quality of optimal ANC care to maximize the benefit obtained from the care.

## Data Availability

The raw data supporting the conclusions of this article will be made available by the authors per the guidance of the data sharing policy of the institute.
