# Supplementary material for: Effect of optimal antenatal care on maternal and perinatal health in Ethiopia
Source: Front Pediatr. 2023 Feb 7;11:1120979. doi: 10.3389/fped.2023.1120979 (PMC9941639; doi:10.3389/fped.2023.1120979)
Supplement: Supplementary file 1 [file Datasheet1.pdf]

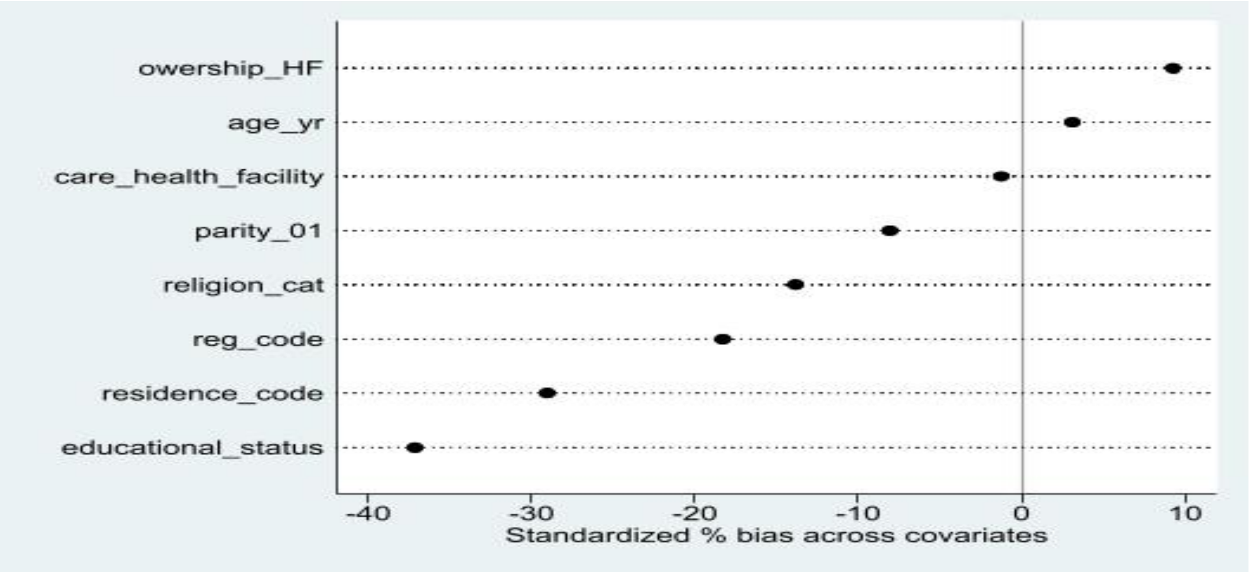

Annex 1: Balance check of propensity score across covariates before matching
(A). Ownership_HF(ownership of health facility), (B) age_yr (maternal age), (C) care_health_faclity (type of health facility), (D) parity_01(maternal parity), (E) religion_cat(religion),(F) residence_code(residence), (G) educational_status(maternal educational status)

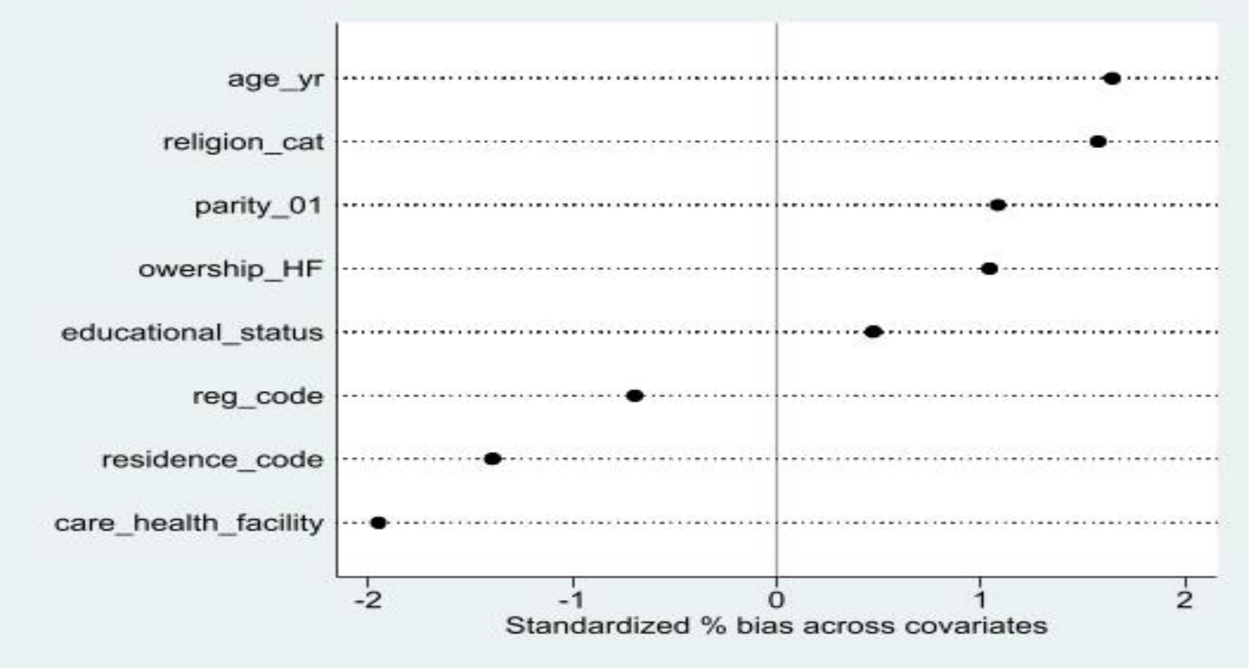

Annex 2: Balance check of propensity score across covariates after matching

(A). ownership_HF (ownership of health facility), (B) age_yr (maternal age), (C) care_health_faclity (type of health facility), (D) parity_01(maternal parity), (E) religion_cat(religion),(F) residence_code(residence), (G) educational_status(maternal educational status)
